# Development and validation of a machine learning–based predictive model for new-onset atrial fibrillation after CABG

**DOI:** 10.3389/fcvm.2026.1783636

**Published:** 2026-04-22

**Authors:** JiaLiang Zheng, ZiRu Li, ShaoTing Sun, MingHao Shi, XiaoBing Lu, DaLin Sun, RuoPu Wang, Song He, ChengZhi Niu, CunTao Yu, Xin Zhang

**Affiliations:** 1 Department of Cardiovascular Surgery, First Affiliated Hospital of Zhengzhou University, Zhengzhou, China; 2 Department of Cardiovascular Surgery, The Second Affiliated Hospital, Zhejiang University School of Medicine, Hangzhou, China

**Keywords:** artificial intelligence, coronary artery bypass grafting, machine learning, new-onset atrial fibrillation, predictive model, SHAP value

## Abstract

**Objective:**

New-onset atrial fibrillation (NOAF) after coronary artery bypass grafting (CABG) is one of the most common post-operative complications in patients undergoing cardiac surgery and is strongly associated with adverse outcomes. We aimed to develop a machine learning (ML) model for predicting NOAF after CABG.

**Method:**

We studied 925 patients who underwent coronary artery bypass grafting (CABG). We randomly split the data into a training set (70%) and a validation set (30%). Our primary outcome was new-onset atrial fibrillation (NOAF) after surgery (i.e., the first episode of atrial fibrillation within 7 days post-operatively in patients who were in sinus rhythm before CABG). We developed eight predictive models based on machine learning algorithms and evaluated their performance using area under the receiver operating characteristic curve (AUC), accuracy, sensitivity, specificity, F1 score, and the Youden index. SHapley Additive exPlanations (SHAP) values were computed and plotted to interpret the contribution of individual features to model predictions.

**Results:**

The incidence of NOAF was 19%. For predicting new-onset atrial fibrillation after CABG, the Gradient Boosting model achieved the highest AUC [0.842 (0.785–0.899)], outperforming the logistic regression model [0.790 (0.722–0.858)]. In addition, AUCs of all machine learning models [0.761–0.842] exceeded those of conventional risk scores, such as CHA2DS2-VASc and HATCH [0.587 (0.55–0.724), 0.62 (0.543–0.697), respectively].

**Conclusion:**

The key features selected by machine learning methods and the resulting predictive models can predict new-onset atrial fibrillation after CABG with reasonable accuracy, which may support exploratory risk stratification and hypothesis generation; external validation is required before clinical implementation.

## Introduction

Coronary artery bypass grafting (CABG) is a cornerstone surgical procedure for the management of complex or diffuse coronary artery disease. By bypassing critically stenotic or occluded coronary segments, CABG improves myocardial perfusion and clinical outcomes. However, new-onset postoperative atrial fibrillation (NOAF) remains one of the most common perioperative complications (1). The reported incidence of NOAF ranges from 20% to 40%, most commonly between 21% and 34%, and it occurs predominantly on post-operative days 1–5, with most cases clustering within 7 days after surgery (2). NOAF is associated with short-term hemodynamic instability, increased risks of stroke and systemic embolism, prolonged hospital and ICU stay, and higher healthcare costs. It is also linked to increased long-term mortality, imposing substantial burdens at both individual and healthcare-system levels (3). Although standardized perioperative management and selective prophylactic strategies (e.g., beta-blockers and amiodarone) can partially reduce the incidence of NOAF, universal prophylaxis is limited by an unfavorable benefit–risk balance and suboptimal cost-effectiveness: most patients remain in sinus rhythm after surgery, and blanket use of pharmacologic or monitoring interventions would inevitably increase unnecessary drug-related adverse effects and economic burden (4). Therefore, individualized risk stratification based on information available in the preoperative and perioperative periods has become a critical clinical issue for precision perioperative management.

In terms of risk prediction tools, traditional clinical scores (such as CHA2DS2-VASc and HATCH) are easy to implement due to their simplicity. However, their overall discrimination is limited, and because they are based primarily on linear relationships, they struggle to capture nonlinear associations and higher-order interactions among predictors (5). Moreover, their performance often degrades when applied to different institutions and patient case mixes.In this study, we aimed to develop and internally validate explainable ML-based prediction models using routinely available perioperative variables to predict NOAF within 7 days after isolated CABG. We also evaluated their discrimination, calibration, and potential clinical utility to inform future external validation and implementation studies.

Unlike previous studies that focused primarily on tree-based models for tabular clinical data, we conducted a head-to-head comparison of eight supervised algorithms spanning linear models, kernel methods, neural networks, and ensemble tree-based approaches and identified Gradient Boosting as the best overall performer. We further implemented a structured feature-selection pipeline combining Boruta screening with LASSO refinement, while retaining inflammatory markers on clinical grounds; robustness was assessed in sensitivity analyses. Finally, we incorporated SHAP-based interpretation to improve clinical transparency and address concerns about “black-box” predictions.

## Materials and methods

### Study design

**Figure 1:**
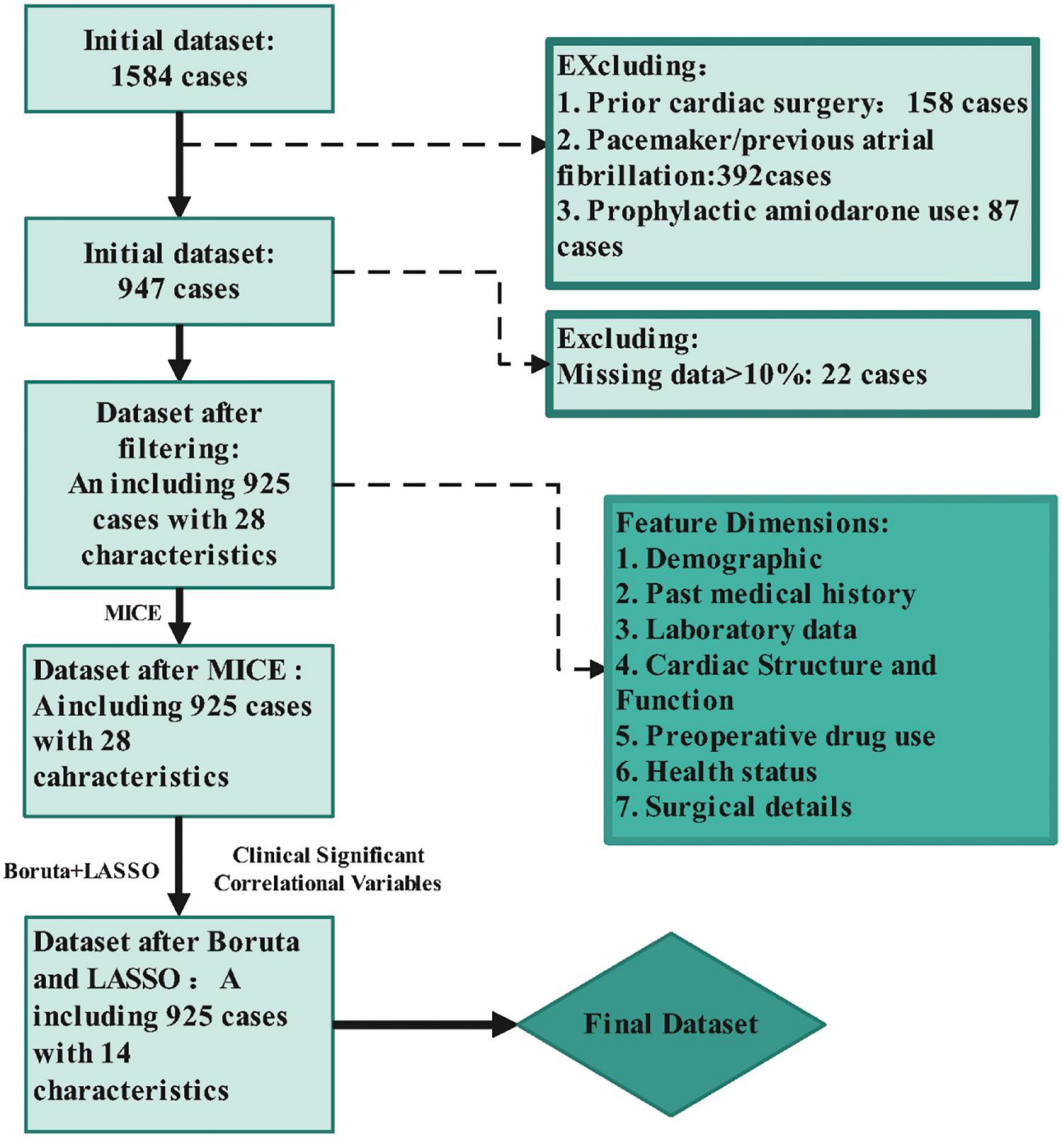
Flowchart of patient selection and data preprocessing for model development. Stepwise exclusion and missing-data filtering of the initial CABG cohort. Imputation and feature selection using MICE, Boruta and LASSO to obtain the final 14-variable dataset.

This study is a single-center, retrospective prognostic investigation. As illustrated in Figure 1, we developed a NOAF prediction model for patients undergoing coronary artery bypass grafting. The model will be developed from single-center real-world data with a limited sample size, with the dataset partitioned 7:3 into a training set and a validation set. Model development and training were performed on the training set, followed by internal validation on the 30% validation set to reduce the risk of overfitting. This study reports results in accordance with the TRIPOD checklist (6). A glossary of terms is provided in Supplementary File S1 and the completed TRIPOD checklist is provided in Supplementary File S2.

### Data resource and study population

This study retrospectively collected data from 925 patients who underwent coronary artery bypass grafting (CABG) at the First Affiliated Hospital of Zhengzhou University between January 2019 and December 2024. New-onset atrial fibrillation (NOAF) was defined as follows: no prior history of atrial fibrillation; postoperative electrocardiogram demonstrating disappearance of P waves replaced by f waves; QRS complexes exhibiting an irregularly irregular rhythm; RR interval variability with episodes lasting longer than 30 s. Furthermore, only atrial fibrillation first documented by continuous cardiac monitoring or 12-lead electrocardiography within 7 days after CABG or prior to hospital discharge (whichever occurred earlier) was counted as an outcome event; atrial fibrillation occurring after discharge was not considered postoperative NOAF (7). Once NOAF was detected, it was jointly confirmed by the cardiac surgeon and the echocardiographer.

Inclusion criteria were: (1) first-time coronary artery bypass grafting (CABG); (2) preoperative electrocardiogram demonstrating sinus rhythm; (3) age ≥ 18 years. Exclusion criteria were: (1) coagulation disorders, valvular heart disease, or other congenital cardiac malformations; (2) prior pacemaker implantation or history of atrial fibrillation; (3) prophylactic perioperative use of amiodarone (Cordarone) (8).

### Missing value handling

Of the initial cohort of 1,584 consecutive patients, we first excluded those with prior cardiac surgery, prior pacemaker implantation, a documented history of atrial fibrillation, or perioperative prophylactic use of amiodarone. This yielded 947 initially eligible cases. We then excluded 22 patients in whom candidate predictor variables had >10% missing data, leaving 925 cases comprising an analysis cohort with 28 baseline variables.Among the remaining 925 samples, the proportion of missing data was <10% for all variables; detailed missing rates are provided in Supplementary File S1. After applying inclusion and exclusion criteria, the analysis cohort (*n* = 925) was first split into a training set (70%) and a validation set (30%) using stratified sampling with a fixed random seed. For both the training and validation sets, the missing rates of candidate variables with <10% missing data are summarized in Supplementary File S1. Missing data were then imputed within the training set only using multiple imputation by chained equations (MICE) (9).All candidate predictors and the outcome (NOAF) were included in the imputation model to generate five imputed training datasets, which were pooled according to Rubin's rules. The imputation procedure was not fit using the validation set. All subsequent feature selection (Boruta and LASSO), model training, and hyperparameter tuning were performed strictly in the training set, and the validation set was used only for final internal evaluation.

### Sample size and feasibility assessment

The study group consists of all CABG patients from our center who met inclusion and exclusion criteria between 2019 and 2024; we did not artificially limit sample size to avoid selection bias. To assess the statistical feasibility of model development, we applied the 10 events-per-variable (10 EPV) principle (10). Fourteen predictors were selected with 176 NOAF events, yielding an EPV of around 12.6, which is the equivalent of the standard “10 events per variable”, and is sufficient to support multi-variable modeling. We applied class weighting only to the training set to address class imbalance in our study, aiming to improve the model's ability to detect positive events while avoiding data leakage. The whole dataset was split 7:3 into training and validation sets. We used 5-fold cross-validation and tuning regularization parameters to reduce overfitting risk resulting from reduced number of events for training sets and model discrimination and calibration in 30% validation cohort.

### Study variables

Based on an analysis of prior studies and expert consensus, and with an emphasis on clinical feasibility and granularity, we included the following seven domains of features in this study (11). In total, we collected 28 predictive features divided into seven groups: demographic characteristics, medical history, laboratory tests, cardiac structural and functional assessments, preoperative medication use, surgery-related information, and general health status (12). (1) Demographics: age, sex, body mass index (BMI). (2) Medical history: hypertension, diabetes mellitus, chronic obstructive pulmonary disease, prior cerebrovascular disease, history of myocardial infarction. (3) Laboratory tests: hemoglobin, creatinine, estimated glomerular filtration rate, potassium, blood sodium level, thyroid-stimulating hormone (TSH), free thyroxine (freeT4), N-terminal pro–B-type natriuretic peptide (NT-proBNP), neutrophil-to-lymphocyte ratio (NLR), C-reactive protein, fibrinogen, albumin. (4) Cardiac structural and functional assessments: left ventricular ejection fraction (LVEF), left atrial diameter. (5) Preoperative medication use: preoperative *β*-blocker use, preoperative use of angiotensin-converting enzyme inhibitors or angiotensin receptor blockers. (6) Surgery-related information: number of grafted vessels, duration of surgery. (7) General health status: nutritional assessment (NRS2002), smoking history (13).

We focused on variables that are routinely available and consistently documented in real-world practice to maximize feasibility of implementation. More granular intraoperative variables (e.g., cardiopulmonary bypass details, cardioplegia strategy, and intraoperative hemodynamics) were not included as candidate predictors because they were not recorded in a sufficiently standardized manner across the retrospective dataset for reliable model development.

### Statistical methods

Continuous variables were presented as mean ± standard deviation or median (interquartile range) according to their distribution, and categorical variables were expressed as counts (percentages). Normally distributed continuous variables were compared using the *t*-test, non-normally distributed variables using the Mann–Whitney *U*-test, and categorical variables using Pearson's *χ*² test. Two-sided *P* < 0.05 was considered statistically significant. We assessed multicollinearity among the variables included in the model by calculating variance inflation factors (VIFs) and performing Pearson correlation analyses; the relevant figures and tables are provided in Supplementary File S1 However, univariate comparisons were used only for descriptive analysis and not as the sole criterion for inclusion in models; final feature selection relied on Boruta and LASSO. All analysis were performed in R (4.4.2).

**Figure 2:**
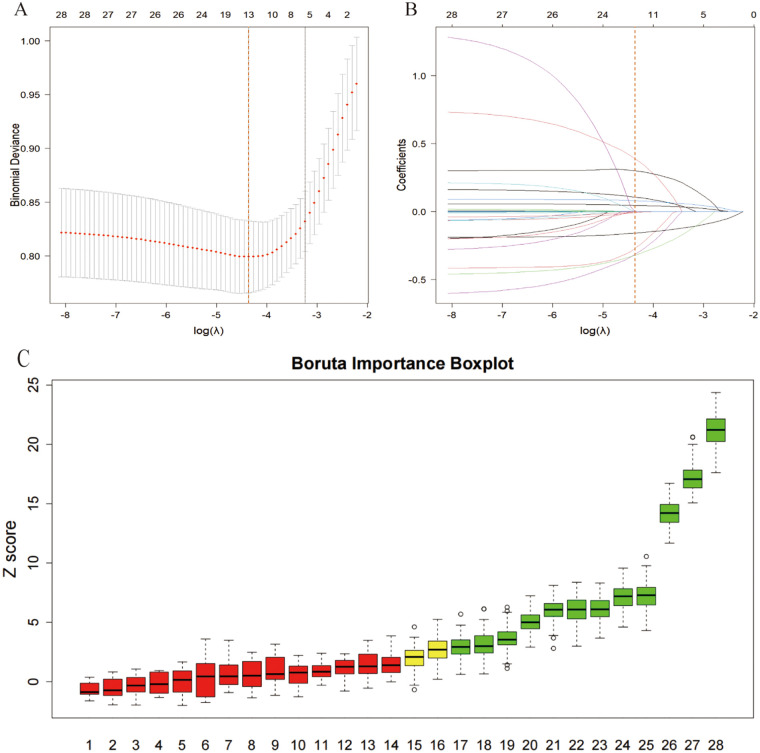
Variable selection using LASSO and boruta. **(A)** cross-validation curve of binomial deviance vs. log **(A)**, indicating the optimal A. **(B)** Coefficient paths across log **(A)**, showing shrinkage and retention of predictors. **(C)** Boruta importance boxplot ranking variables by Z-score and selection status.

Patient data were randomly split into training sets and validation sets in 7:3 ratio, stratified sampling to ensure similar NOAF incidence between the two groups, fixed random seed for reproducibility. Feature selection, model training, and hyperparameter tuning were performed only on training sets, validation set was reserved for internal validation and generalization performance evaluation.To prevent information leakage, the validation set was held out throughout preprocessing and model development and was accessed only once for final internal validation. Feature selection was performed in three steps: “Boruta-based preliminary screening, LASSO-based refinement and forced clinical inclusion”(Figure 2). First, Boruta algorithm based on random forests was used to find important variables.” Next, LASSO regression was applied to further reduce features in the training set, retaining variables with nonzero regression coefficients at the *λ* value corresponding to minimum deviance (14). Finally, informed by prior literature and clinical expertise, C-reactive protein (CRP) and the neutrophil-to-lymphocyte ratio (NLR) were predefined as clinically critical indicators and were forced into the final model even if not selected by LASSO (15). For sensitivity analysis, we repeated model training and evaluation without forcing CRP and NLR into the final feature set, and the performance comparison was reported in Supplementary File S1. All predictors were routinely available perioperative variables, selected to facilitate future integration into electronic health record (EHR) systems for automated risk estimation in patients scheduled for CABG.

Within the unified caret framework, eight supervised learning models were developed: least absolute shrinkage and selection operator (LASSO), discriminant analysis, logistic regression, support vector machine with radial basis function kernel, gradient boosting, Naive Bayes, adaptive boosting, and neural network.For scale-sensitive algorithms (e.g., SVM-RBF, neural network, and LASSO), continuous predictors were centered and scaled (z-scores) using parameters estimated from the training set only, and the same transformation was applied to the validation set to avoid information leakage; tree-based models were trained using predictors on their original scales. Categorical predictors were encoded as binary indicators (0/1) where applicable. Hyperparameters for each model were tuned in the training set using 5-fold cross-validation, with the area under the receiver operating characteristic curve (AUC) as the primary optimization metric (16). In both the training and validation sets, predicted NOAF probabilities were dichotomized using a prespecified default cutoff of 0.5 to derive classification outcomes and to report confusion-matrix-based metrics consistently across models. In clinical practice, the optimal decision threshold should be determined according to the local clinical context and the balance between potential benefits and harms, ideally through prospective evaluation.To avoid over-reliance on a single cutoff, we also assessed threshold-independent discrimination using AUC, and evaluated potential clinical utility across a wide range of threshold probabilities using decision curve analysis. Model discrimination was comprehensively evaluated using AUC, accuracy, Cohen's kappa, sensitivity, specificity, positive predictive value, negative predictive value, F1 score, and Youden's index. ROC curves and AUC with 95% confidence intervals were generated using the pROC package, with 95% CIs calculated by the DeLong method, and were plotted to further assess model performance (17).

**Figure 3:**
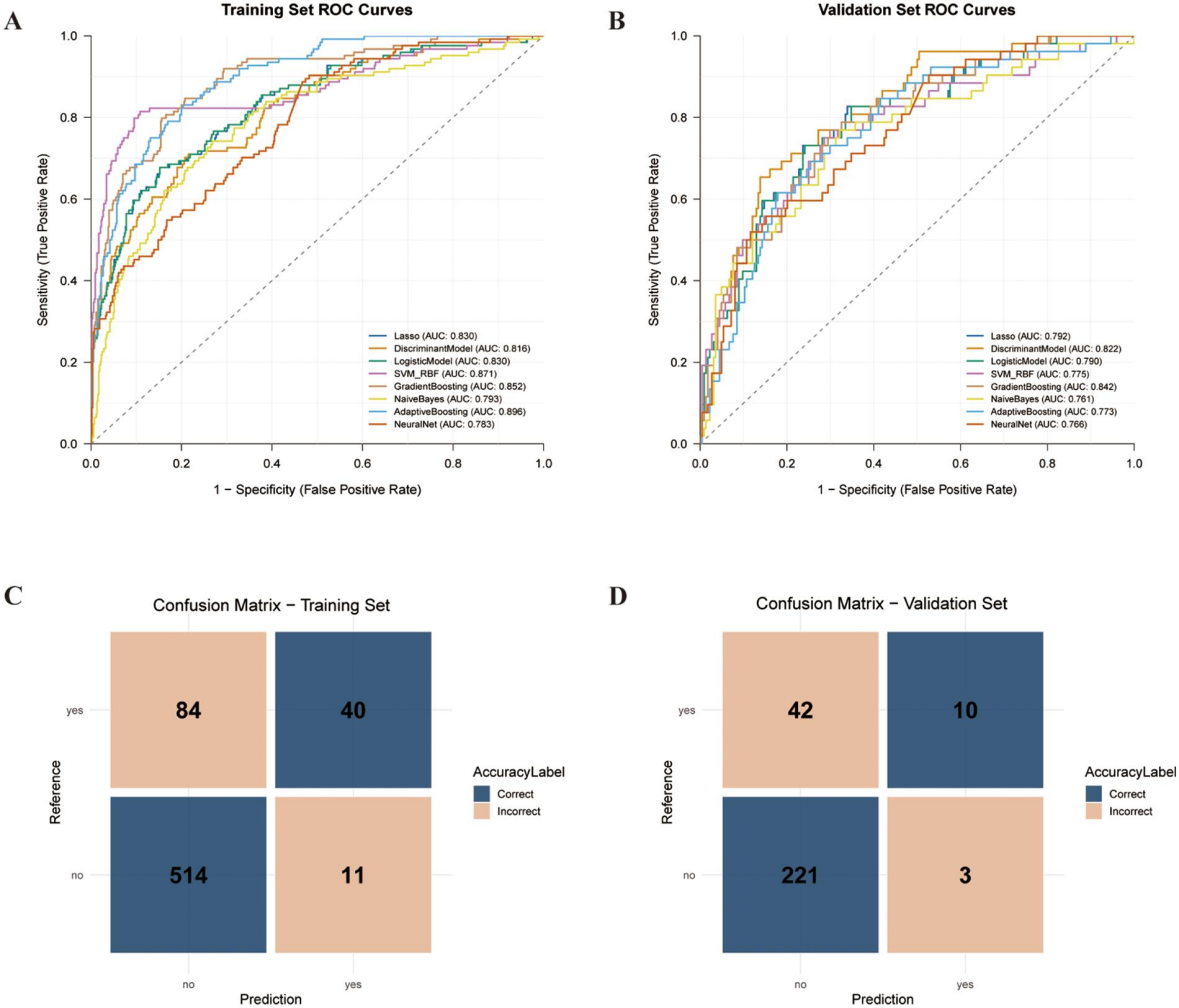
Discrimination of eight machine-learning models and performance of the best classifier. **(A)** ROC curves of all models in the training set. **(B)** ROC curves in the validation set. **(C)** Confusion matrix of the Gradient Boosting model in the training set. **(D)** Confusion matrix of the Gradient Boosting model in the validation set.

To assess calibration of predicted probabilities, samples in the training set were divided into ten groups according to deciles of predicted probability, and a calibration curve of predicted probability vs. observed NOAF incidence was plotted. Based on equal-frequency binning results, predicted probabilities were nonparametrically calibrated using isotonic regression, and the calibration effect was quantified by comparing Brier scores before and after calibration. Decision curve analysis (DCA) was used to compute net benefit across threshold probabilities from 0.01 to 0.99 and to compare these with “treat-all” and “treat-none” strategies to evaluate the potential clinical utility of different models. The model with the highest AUC in the validation set was designated the “optimal model,” and its confusion matrices were plotted for both the training and validation sets to display the distributions of true positives, true negatives, false positives, and false negatives (Figures 3–D).

For the optimal model we computed and plotted SHAP values to show how individual risk factors contribute to the model predictions, as well as how each predictor relates to NOAF risk. We also used shapviz to generate feature importance ranking, SHAP beeswarm plots, and dependence plots for key variables (Figures 5A,B).

## Results

### Baseline characteristics of the patients

We initially collected data from 1,584 patients. After applying inclusion and exclusion criteria and removing records with substantial missing data, 925 cases were included in our study. The dataset was split 7:3, yielding 649 patients in the training set and 276 patients in the validation set. In the training set, 124 patients (19%) developed postoperative NOAF, while 525 patients (81%) maintained sinus rhythm postoperatively (Table 1). Detailed demographic characteristics and clinical variables for the training cohort used to develop the model are provided in Supplementary File S1.

**Table 1:** Baseline characteristics of training set.

Variables	Total(n=649)	Non_NOAF(n=525)	NOAF(n=124)	P-value
NRS2002				<0.001
0	522（80.4%）	439（83.6%）	83（66.9%）	
1	109（16.8%）	74（14.1%）	35（28.2%）	
2	13（2.0%）	9（1.7%）	4（3.2%）	
3	5（0.8%）	3（0.6%）	2（1.6%）	
Age(years)	63.00 (57.00–69.00)	62.00 (56.00–68.00)	68.00 (59.75–72.00)	<0.001
CRP(mg/L)	1.72 (0.77–4.76)	1.84 (0.79–4.84)	1.41 (0.69–4.41)	0.602
Creatinine(umol/L)	74.00 (64.00–88.00)	73.00 (63.00–86.00)	79.50 (68.50–95.12)	0.001
FT4（pmol/L）	11.87 (10.45–13.55)	11.74 (10.46–13.35)	12.38 (10.38–14.20)	0.089
Fibrinogen(g/L)	3.08 (2.65–3.63)	3.09 (2.65–3.63)	3.04 (2.69–3.68)	0.834
Hemoglobin (g/L)	132.00 (120.00–144.00)	132.00 (121.00–144.00)	131.00 (118.00–144.00)	0.468
Potassium (mmol/L)	4.20 (3.90–4.45)	4.21 (3.94–4.46)	4.10 (3.70–4.42)	0.007
LAD(mm)	37.00 (33.00–40.00)	37.00 (33.00–40.00)	40.00 (36.00–45.00)	<0.001
LVEF(%)	60.00 (51.00–63.00)	60.00 (52.00–63.00)	58.00 (47.00–62.00)	0.010
NLR	2.25 (1.74–3.09)	2.23 (1.72–3.07)	2.55 (1.81–3.24)	0.066
NT-proBNP (pg/mL)	277.15 (102.00–845.46)	236.00 (90.44–746.10)	496.00 (208.75–1345.00)	<0.001
Sodium(mmol/L)	141.00 (139.00–143.00)	141.00 (139.60–143.00)	139.00 (136.00–142.00)	<0.001
TSH(mIU/L)	2.34 (1.45–3.55)	2.47 (1.56–3.73)	1.79 (1.02–2.86)	<0.001
Number of grafts	3.00 (3.00–4.00)	3.00 (3.00–4.00)	3.00 (2.00–4.00)	<0.001
eGFR(mL/min/1.73m²)	90.41 (77.39–98.77)	91.20 (80.26–99.86)	82.84 (64.80–94.39)	<0.001
operation_time(min)	275.00 (240.00–320.00)	275.00 (240.00–316.00)	270.00 (227.50–341.25)	0.715
ACEI/ARB use, n(%)				0.212
no	573 (88.3%)	459 (87.4%)	114 (91.9%)	
yes	76 (11.7%)	66 (12.6%)	10 (8.1%)	
Beta-Blocker use,n(%)				0.149
no	603 (92.9%)	492 (93.7%)	111 (89.5%)	
yes	46 (7.1%)	33 (6.3%)	13 (10.5%)	
COPD, n (%)				1.000
no	639 (98.5%)	517 (98.5%)	122 (98.4%)	
yes	10 (1.5%)	8 (1.5%)	2 (1.6%)	
Diabetes, n (%)				0.456
no	413 (63.6%)	330 (62.9%)	83 (66.9%)	
yes	236 (36.4%)	195 (37.1%)	41 (33.1%)	
Gender, n (%)				0.885
female	172 (26.5%)	138 (26.3%)	34 (27.4%)	
male	477 (73.5%)	387 (73.7%)	90 (72.6%)	
Hypertension, n (%)				0.950
no	247 (38.1%)	199 (37.9%)	48 (38.7%)	
yes	402 (61.9%)	326 (62.1%)	76 (61.3%)	
MI_history, n (%)				0.008
no	550 (84.7%)	455 (86.7%)	95 (76.6%)	
yes	99 (15.3%)	70 (13.3%)	29 (23.4%)	
Stroke_TIA, n (%)				0.053
no	499 (76.9%)	395 (75.2%)	104 (83.9%)	
yes	150 (23.1%)	130 (24.8%)	20 (16.1%)	
smoking, n (%)				0.456
no	339 (52.2%)	270 (51.4%)	69 (55.6%)	
yes	310 (47.8%)	255 (48.6%)	55 (44.4%)	

As shown in Figure 1, after completing data preprocessing, we applied the Boruta algorithm and identified 13 features with statistically significant differences, including Age, Creatinine, eGFR, K_pre, bridge_counts, and operation_time. These features were further subjected to LASSO regression analysis, which under the optimal penalty identified 12 nonzero coefficients: Age, Creatinine, eGFR, potassium (preoperative), sodium(preoperative), TSH, NT-proBNP, Alb, LAD, preoperative ACEI/ARB use, number of grafted vessels, and operative duration. These 12 features will be incorporated into various machine learning algorithms to construct predictive models. In addition, based on the clinical experience of cardiothoracic surgeons, two inflammatory markers—CRP and NLR (neutrophil to lymphocyte ratio)—were also included in the models for analysis.

### Development and performance of the NOAF model

In the machine learning analysis of this study, 925 patients who underwent CABG were included, of whom 176 (19.0%) developed postoperative new-onset atrial fibrillation (NOAF) and 749 (81.0%) maintained sinus rhythm. Based on 28 perioperative variables, we developed and compared eight supervised learning models to predict postoperative NOAF: LASSO logistic regression, linear discriminant analysis, multivariable logistic regression, radial basis function support vector machine (SVM-RBF), gradient boosting (GB), naive Bayes, AdaBoost, and a feedforward neural network. Model performance was evaluated on both the training set and an independent validation set using AUC, accuracy, sensitivity, specificity, F1 score, and the Youden index.All evaluation metrics for the models used in this study are provided in the Supplementary File S1.Moreover, In sensitivity analyses excluding CRP and NLR, the Gradient Boosting model showed lower overall performance in the validation set. Specifically, adding CRP and NLR improved AUC (0.842 vs. 0.802), accuracy (0.772 vs. 0.753), sensitivity (0.780 vs. 0.308), F1 score (0.570 vs. 0.427), and Youden index (0.550 vs. 0.276), with a modest trade-off in specificity (0.770 vs. 0.813). The corresponding performance comparison figures are provided in Supplementary File S1.

Overall, the algorithms demonstrated fair-to-good discrimination for NOAF on the validation set, with AUCs ranging from 0.761 to 0.842 (Figure 3B). Traditional linear models (LASSO, linear discriminant analysis, and logistic regression) achieved validation-set AUCs of 0.790–0.822, accuracy of 0.754–0.823, and F1 scores of approximately 0.528–0.581. SVM-RBF and AdaBoost exhibited slightly higher validation-set AUCs than the linear models (0.775 and 0.773, respectively), but showed trade-offs between sensitivity and specificity. Naive Bayes and neural networks performed relatively weaker or less balanced overall (validation-set AUCs of 0.761 and 0.766, respectively, with F1 scores of approximately 0.488–0.504).

Among all candidate algorithms, the Gradient Boosting (GB) model exhibited the best overall predictive performance (Figures 3A,B). In the training set, GB achieved an AUC of 0.852 (95% CI 0.820–0.883), accuracy of 0.823, sensitivity of 0.798, specificity of 0.844, F1 score of 0.649, and the highest Youden index (0.620), indicating strong discriminative ability without evident overfitting. In the independent validation set, GB maintained the highest AUC at 0.842 (95% CI 0.785–0.899), with accuracy 0.772, sensitivity 0.780, specificity 0.770, F1 score 0.570, and Youden index 0.550, showing consistent performance in internal validation. Compared with conventional postoperative atrial fibrillation risk scoring models for CABG populations (18), the GB model not only exhibited a substantially higher AUC but also achieved a better balance between sensitivity and specificity, showing promise for more accurately identifying patients at high risk of postoperative NOAF and thereby supporting intensified monitoring or preventive interventions (19).

Decision curve analysis suggested that, across a clinically reasonable range of threshold probabilities, the proposed ML models could provide a higher net benefit than “treat-all” and “treat-none” strategies, supporting their potential role in risk-stratified prophylaxis or intensified monitoring rather than universal interventions (Figure 5B) (20). Calibration curves show that the predicted risk of most models is well proportional to the NOAF observed in the validation set (Figure 5A), and gradient boosting model is more correlated in the intermediate range, which suggested it is reliable as postoperative NOAF risk stratification tool (21). Quantitatively, isotonic calibration slightly improved the Brier score of the Gradient Boosting model in the validation set, decreasing from 0.1408 to 0.1351, indicating improved overall probabilistic accuracy.

We also benchmarked our machine learning models against established clinical risk scores used for AF risk stratification. In our cohort, the CHA₂DS₂-VASc and HATCH scores achieved AUCs of 0.587 (95% CI 0.550–0.724) and 0.620 (95% CI 0.543–0.697), respectively, which were substantially lower than those of all eight ML-based classifiers (AUC range 0.761–0.842).Notably, the Gradient Boosting (GB) model outperformed traditional risk scores for identifying NOAF after CABG.

Finally, the tree-based ensemble GB algorithm performed better in this study than other traditional models and other machine learning techniques. This suggests that modern machine learning techniques (especially ensemble methods capable of capturing complex nonlinear feature relationships) can offer significant advantages in risk stratification and prediction of NOAF after CABG.

### Model interpretability

**Figure 4:**
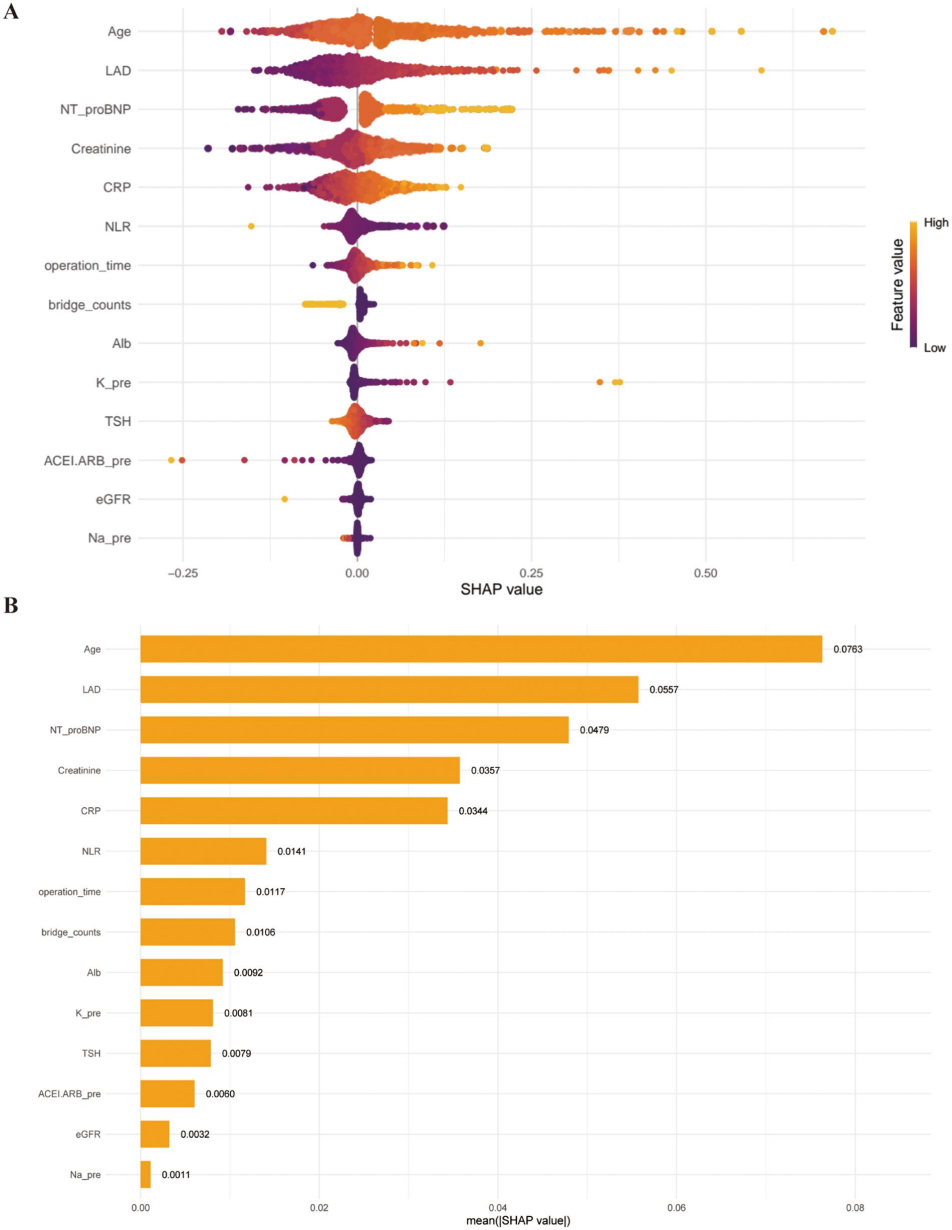
SHAP-based interpretation of the optimal predictive model.**(A)** Beeswarm plot showing the distribution of SHAP values for eachfeature; positive SHAP values indicate increased predicted risk, whereas negative values indicate reduced risk. Color intensity reflects feature magnitude (higher values in warmer colors).**(B)** Bar plot of mean absolute SHAP values summarizing the relative importance ofthe 14 variables, with Age, LAD, and NT-proBNP contributing most to the model.

Our SHAP-based interpretability analysis showed that the Gradient Boosting model identified a set of clinically plausible predictors of NOAF after CABG in Figure 4. Elder age and larger left atrial diameter were the most influential features, showing clear nonlinear positive associations with NOAF risk and underscoring the importance of preexisting structural remodeling. Inflammatory and nutritional markers (CRP, NLR, albumin), NT-proBNP, operative duration, and renal function (captured predominantly by creatinine rather than eGFR) also emerged as important contributors, reflecting systemic inflammation, hemodynamic stress, and cardiorenal risk. Preoperative ACEI/ARB use and higher graft counts were associated with lower predicted NOAF risk in this model. However, these patterns should be interpreted as model-based associations rather than causal effects, given the observational design and the possibility of confounding and treatment/selection biases. Overall, these SHAP patterns reinforce the biological plausibility and clinical interpretability of the model's predictions.

**Figure 5:**
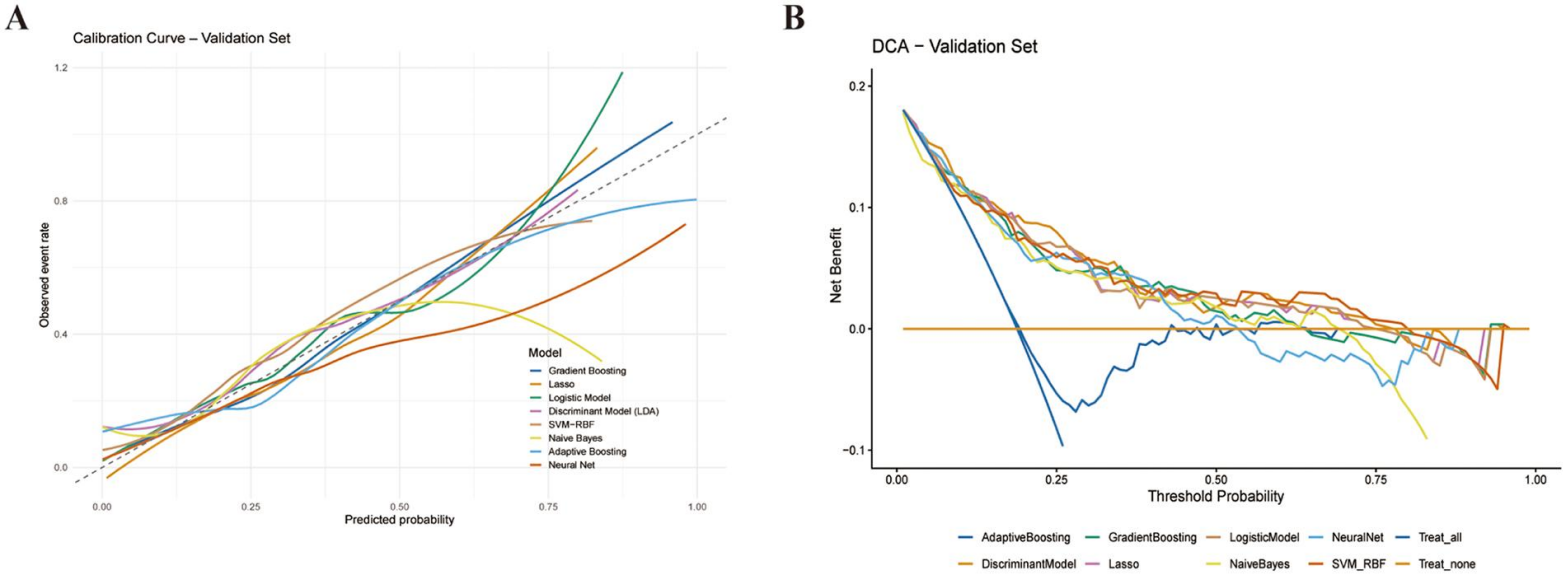
Calibration and clinical utility of the prediction models in the validation set. **(A)** Calibration curves after isotonic recalibration comparing predicted and observedNOAF risk. **(B)** Decision curve analysis showing net benefit across threshold probabilities vs. “treat-all” and “treat-none” strategies.

### Clinical workflow integration

From an implementation standpoint, the model could be deployed as an EHR-integrated risk calculator that auto-populates routinely collected preoperative variables and returns an individualized NOAF risk estimate. Patients could then be triaged into pragmatic risk categories to support local, protocol-driven actions such as enhanced rhythm monitoring, earlier correction of electrolyte abnormalities, and selective consideration of prophylactic therapy. This approach is intended to support perioperative planning without adding substantial bedside workload.

## Discussion

In this single-center cohort of 925 patients with preoperative sinus rhythm undergoing isolated CABG, we developed and internally validated a machine learning–based model to predict new-onset postoperative atrial fibrillation using 14 routinely available perioperative variables. Across a systematic comparison of eight supervised algorithms, overall model performance ranged from fair to good. Among these, the Gradient Boosting model demonstrated superior and consistent discriminative performance, together with an acceptable balance between sensitivity and specificity and satisfactory calibration in the validation cohort.To mitigate concerns regarding the “black box” nature of machine learning approaches, SHAP values were incorporated to facilitate model interpretability and to provide case-level explanations for individual risk estimates (22). This strategy allows clinicians to better understand the contribution of specific features to predicted risk, addressing a key barrier to clinical adoption of machine learning models.While the development pipeline is technically involved, the intended end-user workflow is simple: a limited number of routinely available variables are entered or retrieved, and the system returns a predicted risk together with SHAP-based explanations. We also report decision-curve analysis to illustrate the potential net benefit across clinically plausible thresholds, complementing conventional discrimination metrics.In addition, the integration of decision curve analysis enabled an assessment of potential clinical utility across a range of threshold probabilities, thereby complementing traditional performance metrics and addressing limitations previously reported for conventional regression-based models and established risk scores.Importantly, improved discrimination alone does not automatically translate into clinical benefit. The practical value of the model lies in enabling risk stratification to target preventive strategies (e.g., intensified rhythm surveillance and selective pharmacologic prophylaxis) to patients most likely to develop NOAF, thereby potentially reducing unnecessary exposure and resource use associated with universal prophylaxis. Recent externally validated tools, such as the POLARIS score, prioritize simplicity and generalizability(23). We view our work as complementary. Specifically, we focused on an isolated CABG cohort with a clearly defined 7-day NOAF outcome and used routinely available perioperative variables to support center-level risk stratification. In addition to comparing several supervised learning algorithms within a structured feature-selection framework, we conducted a broader model evaluation beyond discrimination, including calibration assessment, decision curve analysis, and SHAP-based interpretability. Nevertheless, as this study is based on a single-center cohort and has undergone internal validation only, external validation will be necessary before considering broader clinical application.

Consistent with prior literature, SHAP-based interpretability analysis identified elder age and increased left atrial end-diastolic diameter as the two most influential predictors of NOAF following CABG. The beeswarm plot revealed a clear nonlinear positive association between these variables and NOAF risk, indicating that their effects are not strictly linear across the observed range. From a pathophysiological standpoint, aging is closely associated with progressive atrial fibrosis, amyloid deposition, and electrical heterogeneity, all of which contribute to a substrate that facilitates re-entry and atrial arrhythmogenesis (24).Similarly, enlargement of the left atrium reflects chronic pressure overload and long-standing structural remodeling, which may predispose the atria to electrical instability when exposed to perioperative stressors (25). The predominance of these variables in the SHAP analysis suggests that patients at highest risk of NOAF already possess an unfavorable atrial structural substrate prior to surgery. This observation aligns with previous findings, including those reported by Zhang et al. (26), highlighting the importance of cardiac structural parameters in postoperative atrial fibrillation risk stratification.

Beyond structural factors, several biomarkers reflecting inflammatory status and nutritional reserve—including CRP, NLR, and serum albumin—also emerged as important contributors to NOAF risk in our model (27). These findings lend further support to the inflammation hypothesis of postoperative atrial fibrillation, whereby surgical trauma elicits a systemic inflammatory response that promotes oxidative stress and atrial electrical remodeling. Elevated CRP and NLR values were associated with progressively higher SHAP values, indicating a strong and consistent shift toward increased NOAF risk. This is in agreement with recent machine learning–based studies, such as that by Turkkolu et al (28), which identified inflammatory cell indices as key predictors of POAF (29).

Operative duration likewise demonstrated a sustained positive association with NOAF risk, with SHAP values increasing in a dose–response–like manner as operative time lengthened. Prolonged operative time may act as a composite surrogate for multiple injurious mechanisms, including ischemia–reperfusion injury, prolonged inflammatory activation, and heightened sympathetic stimulation, all of which are known to facilitate atrial electrical instability (30). In contrast, higher serum albumin levels were associated with negative SHAP values, suggesting a protective effect. As a marker of nutritional status and physiological reserve, albumin may reflect an enhanced capacity to tolerate surgical stress and inflammatory burden, thereby reducing susceptibility to postoperative atrial fibrillation (31).

In this study, among renal function–related indicators, creatinine contributed most to the prediction of NOAF, whereas the contribution of eGFR to the outcome events was comparatively limited. Renal dysfunction is well recognized as a surrogate for vascular calcification, fluid retention, and a proinflammatory state (32). However, in our model the SHAP importance of creatinine was markedly higher than that of eGFR. This suggests that risk signals along the “cardiorenal” axis are primarily captured by creatinine, while eGFR offers only limited incremental information. Considering that, compared with traditional regression models, SHAP emphasizes marginal contributions and nonlinear relationships: when upstream factors highly correlated with eGFR—such as age and creatinine—are included in the model, the incremental contribution of eGFR becomes relatively small. A “creatinine-dominant, eGFR-secondary” pattern is therefore to be expected. Based on these findings, within the “renal dysfunction” section we describe it as “a risk background primarily reflected by creatinine with eGFR providing ancillary information,” in order to avoid overinterpretation of the model outputs (33).

Preoperative use of ACEI/ARB in our cohort exhibited a protective effect (higher values for negative SHAP contributions). Although some literature doubts their role in outcomes, our machine learning model shows that RAAS blockade only provides modest net benefit in nonlinear interactions (34). Bridge_counts(the number of grafted vessels) showed an inverse association with predicted NOAF risk in the SHAP analysis. This finding may reflect residual confounding or surgical selection (e.g., patient complexity, coronary anatomy, surgeon preference, perioperative management intensity), rather than a direct protective effect of graft number itself. Therefore, this feature should not be interpreted as evidence that increasing graft counts would reduce NOAF risk (35).

### Limitation

Several limitations of this study should be acknowledged. First, this was a single-center, retrospective cohort study. Although consecutive CABG patients were included and the analysis was restricted to individuals with preoperative sinus rhythm, data quality and variable completeness remained dependent on routine clinical documentation, introducing the potential for selection and information bias.Accordingly, our model should be considered a preliminary, single-center tool intended to generate hypotheses and support future multi-center validation rather than to replace established externally validated scores at this stage. In addition, model development and validation were confined to a single institution, and external validation was not performed. As a result, the generalizability of the model to other clinical settings cannot be assumed and requires confirmation in independent, multi-center cohorts.Differences in case-mix, perioperative care pathways, and rhythm-monitoring practices across hospitals may affect both the baseline NOAF rate and model performance. Therefore, external validation and, if needed, model recalibration are required before broader clinical use. Furthermore, internal validation in this study was based on a single stratified 70/30 train–validation split with a modest number of NOAF events. Although 5-fold cross-validation was applied within the training set for hyperparameter tuning and model selection, this approach may produce more variable performance estimates than repeated or nested cross-validation applied to the full cohort. Accordingly, the reported discrimination and calibration metrics should be interpreted as approximate estimates of internal performance. Further analyses using repeated or nested cross-validation, and—most importantly—external validation in independent cohorts, will be required to obtain more stable and generalizable performance estimates.

Second, although we incorporated routinely recorded intraoperative surrogates (operative duration and number of grafted vessels), more granular intraoperative parameters (e.g., cardiopulmonary bypass and cardioplegia details, temperature management, transfusion, and vasoactive support) were not included. In this retrospective dataset, these variables showed substantial missing data and heterogeneous documentation formats, which limited their reliable standardization for model development. Moreover, because the model is intended to support timely perioperative risk stratification, we prioritized predictors that are consistently available at admission or immediately before surgery to facilitate early preventive planning.

Third, NOAF was ascertained from in-hospital documentation based on continuous monitoring and/or 12-lead ECG within 7 days after CABG or before discharge, whichever occurred earlier. Because uninterrupted telemetry was not uniformly available for all patients throughout the entire risk window, short-lived or asymptomatic AF episodes may have been missing. Such outcome misclassification is difficult to fully address retrospectively and may attenuate the estimated model performance. Future prospective studies with standardized continuous rhythm monitoring and multi-center external validation will be important to improve outcome ascertainment and to confirm the model's transportability.

Moreover, all predictors incorporated into the model were derived from routinely available clinical variables and standard laboratory measurements. While this design enhances practical applicability, it also limits the inclusion of potentially informative data. Variables related to atrial structural remodeling, myocardial fibrosis, genetic predisposition, multi-omics profiles, and advanced inflammatory biomarkers were not available and therefore not assessed. The absence of these factors may have led to an underestimation of the contribution of certain pathophysiological pathways to NOAF development.

Finally, although the proposed machine learning model demonstrated improved performance compared with existing clinical risk scores such as CHA2DS2-VASc and HATCH within this cohort, the retrospective nature of the study precludes conclusions regarding direct clinical benefit. Prospective studies and randomized controlled trials are needed to evaluate the impact of model-guided risk stratification on clinical decision-making and patient outcomes in real-world settings.

## Conclusion

Our results show that machine learning predictive models significantly outperform standard scores such as CHA2DS2-VASc and HATCH in forecasting new-onset atrial fibrillation after coronary artery bypass grafting. These findings suggest that an explainable ensemble model can improve perioperative NOAF risk stratification after isolated CABG compared with conventional scores. However, external validation and prospective impact studies are needed prior to routine clinical adoption. Future work should better understand how feature profiles between different patient groups influence NOAF onset timing.

## Data Availability

The datasets presented in this study can be found in online repositories. The names of the repository/repositories and accession number(s) can be found in the article/Supplementary Material.
