# Supplementary material for: Development and validation of a machine learning–based predictive model for new-onset atrial fibrillation after CABG
Source: Front Cardiovasc Med. 2026 Apr 22;13:1783636. doi: 10.3389/fcvm.2026.1783636 (PMC13143740; doi:10.3389/fcvm.2026.1783636)
Supplement: Supplementary file 1 [file Table1.docx]

Supplementary Material

# 1. supplementary Figures and Tables

1.1 VIF and pearson test


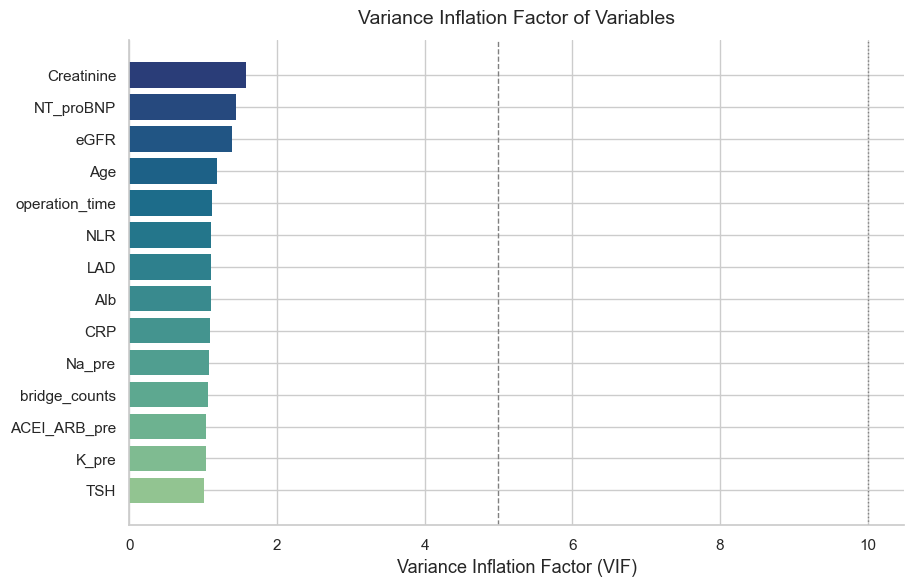




1.2 The evaluation metrics of ML models

| **Model** | **Set** | **Youden** | **AUC (95%CI)** | **Accuracy (95%CI)** | **Sensitivity (95%CI)** | **Specificity (95%CI)** | **F1 (95%CI)** |
| --- | --- | --- | --- | --- | --- | --- | --- |
| Lasso | Train | 0.525 | 0.830 (0.788–0.872) | 0.815 (0.787–0.846) | 0.677 (0.595–0.759) | 0.848 (0.817–0.878) | 0.583 (0.513–0.651) |
|  | Test | 0.494 | 0.792 (0.724–0.859) | 0.757 (0.710–0.808) | 0.731 (0.600–0.851) | 0.763 (0.708–0.816) | 0.532 (0.423–0.622) |
| Discriminant Model | Train | 0.494 | 0.816 (0.773–0.859) | 0.770 (0.740–0.806) | 0.710 (0.627–0.787) | 0.785 (0.750–0.822) | 0.542 (0.474–0.607) |
|  | Test | 0.516 | 0.822 (0.762–0.883) | 0.823 (0.779–0.866) | 0.654 (0.512–0.784) | 0.862 (0.811–0.906) | 0.581 (0.459–0.687) |
| Logistic Model | Train | 0.525 | 0.830 (0.788–0.872) | 0.815 (0.787–0.846) | 0.677 (0.595–0.759) | 0.848 (0.817–0.878) | 0.583 (0.513–0.651) |
|  | Test | 0.490 | 0.790 (0.722–0.858) | 0.754 (0.703–0.804) | 0.731 (0.600–0.851) | 0.759 (0.701–0.810) | 0.528 (0.418–0.618) |
| SVM-RBF | Train | 0.706 | 0.872 (0.828–0.915) | 0.877 (0.852–0.901) | 0.814 (0.744–0.883) | 0.891 (0.865–0.917) | 0.716 (0.653–0.775) |
|  | Test | 0.464 | 0.775 (0.700–0.851) | 0.721 (0.670–0.772) | 0.750 (0.617–0.860) | 0.714 (0.659–0.773) | 0.503 (0.403–0.592) |
| Gradient Boosting | Train | 0.620 | 0.852 (0.820–0.883) | 0.823 (0.804–0.864) | 0.798 (0.723–0.865) | 0.844 (0.810–0.876) | 0.649 (0.581–0.712) |
|  | Test | 0.550 | ⭐ 0.842 (0.785–0.899) | 0.772 (0.723–0.819) | 0.780 (0.660–0.890) | 0.770 (0.720–0.830) | 0.570 (0.470–0.660) |
| Naive Bayes | Train | 0.473 | 0.793 (0.746–0.839) | 0.733 (0.698–0.767) | 0.742 (0.661–0.820) | 0.731 (0.694–0.770) | 0.515 (0.450–0.581) |
|  | Test | 0.448 | 0.761 (0.685–0.838) | 0.696 (0.641–0.750) | 0.769 (0.644–0.875) | 0.679 (0.615–0.737) | 0.488 (0.387–0.577) |
| Adaptive Boosting | Train | 0.627 | 0.896 (0.868–0.924) | 0.803 (0.770–0.834) | 0.831 (0.760–0.894) | 0.796 (0.758–0.828) | 0.617 (0.552–0.680) |
|  | Test | 0.438 | 0.773 (0.704–0.842) | 0.736 (0.685–0.786) | 0.692 (0.555–0.817) | 0.746 (0.690–0.798) | 0.497 (0.387–0.587) |
| Neural Net | Train | 0.421 | 0.783 (0.740–0.827) | 0.592 (0.555–0.630) | 0.903 (0.855–0.951) | 0.518 (0.476–0.559) | 0.458 (0.405–0.511) |
|  | Test | 0.406 | 0.766 (0.698–0.835) | 0.794 (0.743–0.841) | 0.558 (0.411–0.691) | 0.848 (0.800–0.890) | 0.504 (0.378–0.607) |

1.3 The glossary of terms

| Category | Variable | Type | Unit | Description |
| --- | --- | --- | --- | --- |
| I. Demographics | Age | Continuous | years | Patient age, generally recorded as an integer |
| I. Demographics | Sex | Binary | — | 0 = female, 1 = male |
| I. Demographics | BMI | Continuous | kg/m² | Body mass index, calculated as weight (kg) / height² (m²) |
| II. Medical history | Hypertension | Binary | — | 1 = yes, 0 = no |
| II. Medical history | Diabetes | Binary | — | 1 = yes, 0 = no |
| II. Medical history | COPD | Binary | — | 1 = yes, 0 = no |
| II. Medical history | Stroke_TIA | Binary | — | 1 = history of stroke or transient ischemic attack, 0 = no |
| II. Medical history | MI_history | Binary | — | 1 = yes, 0 = no |
| III. Laboratory tests | Hb | Continuous | g/L | Normal range: male 120–160 g/L, female 110–150 g/L |
| III. Laboratory tests | Creatinine | Continuous | μmol/L | Normal range: 44–104 μmol/L (varies by age and sex) |
| III. Laboratory tests | eGFR | Continuous | mL/min/1.73 m² | Normal: ≥90 mL/min/1.73 m² |
| III. Laboratory tests | K_pre | Continuous | mmol/L | Normal range: 3.5–5.5 mmol/L |
| III. Laboratory tests | TSH | Continuous | mIU/L | Normal range: 0.3–4.5 mIU/L |
| III. Laboratory tests | FT4 | Continuous | pmol/L | Normal range: 9–25 pmol/L |
| III. Laboratory tests | NT_proBNP | Continuous | pg/mL | Cardiac biomarker; normal values vary with age |
| III. Laboratory tests | NLR | Continuous | — | Normal value < 3 |
| III. Laboratory tests | CRP | Continuous | mg/L | Normal < 5 mg/L |
| III. Laboratory tests | Fibrinogen | Continuous | g/L | Normal range: 2–4 g/L |
| III. Laboratory tests | Alb | Continuous | g/L | Normal range: 35–55 g/L |
| III. Laboratory tests | Na_pre | Continuous | mmol/L | Normal range: 135–145 mmol/L |
| IV. Cardiac function & imaging | LVEF | Continuous | % | Normal ≥ 55%, indicates preserved systolic function |
| IV. Cardiac function & imaging | LAD | Continuous | mm | Normal ≤ 40 mm (age- and sex-dependent) |
| V. Medication use | BetaBlocker_pre | Binary | — | 1 = preoperative use, 0 = no use |
| V. Medication use | ACEI_ARB_pre | Binary | — | 1 = preoperative ACEI/ARB use, 0 = no use |
| VI. Others | NRS2002 | Continuous | points | Nutritional Risk Screening 2002; ≥3 indicates nutritional risk |
| VI. Others | smoking | Binary | — | 1 = history of smoking, 0 = never smoked |
| VII. Surgery-related | bridge_counts | Continuous | number | Number of bypass grafts |
| VII. Surgery-related | operation_time | Continuous | minutes | Duration of surgery |

1.4 Missing data for each variable

| Variable | All sample | Missing variable | Missing Proportion |
| --- | --- | --- | --- |
| CRP | 925 | 82 | 8.86% |
| NRS2002 | 925 | 70 | 7.57% |
| TSH | 925 | 58 | 6.27% |
| FT4 | 925 | 58 | 6.27% |
| NT_proBNP | 925 | 46 | 4.97% |
| Fibrinogen | 925 | 40 | 4.32% |
| Alb | 925 | 22 | 2.38% |
| LAD | 925 | 20 | 2.16% |
| NLR | 925 | 18 | 1.95% |
| LVEF | 925 | 18 | 1.95% |
| Creatinine | 925 | 17 | 1.84% |
| eGFR | 925 | 17 | 1.84% |
| Hb | 925 | 16 | 1.73% |
| BMI | 925 | 14 | 1.51% |
| K_pre | 925 | 12 | 1.3% |
| Na_pre | 925 | 12 | 1.3% |
| operation_time | 925 | 6 | 0.65% |
| Hypertension | 925 | 4 | 0.43% |
| bridge_counts | 925 | 4 | 0.43% |
| Diabetes | 925 | 3 | 0.32% |
| BetaBlocker_pre | 925 | 3 | 0.32% |
| smoking | 925 | 3 | 0.32% |
| COPD | 925 | 2 | 0.22% |
| Stroke_TIA | 925 | 2 | 0.22% |
| MI_history | 925 | 2 | 0.22% |
| ACEI/ARB_pre | 925 | 2 | 0.22% |
| Patient_ID | 925 | 0 | 0% |
| Age | 925 | 0 | 0% |
| Gender | 925 | 0 | 0% |
| NOAF_outcome | 925 | 0 | 0% |

1.5 Sensitive Analysis


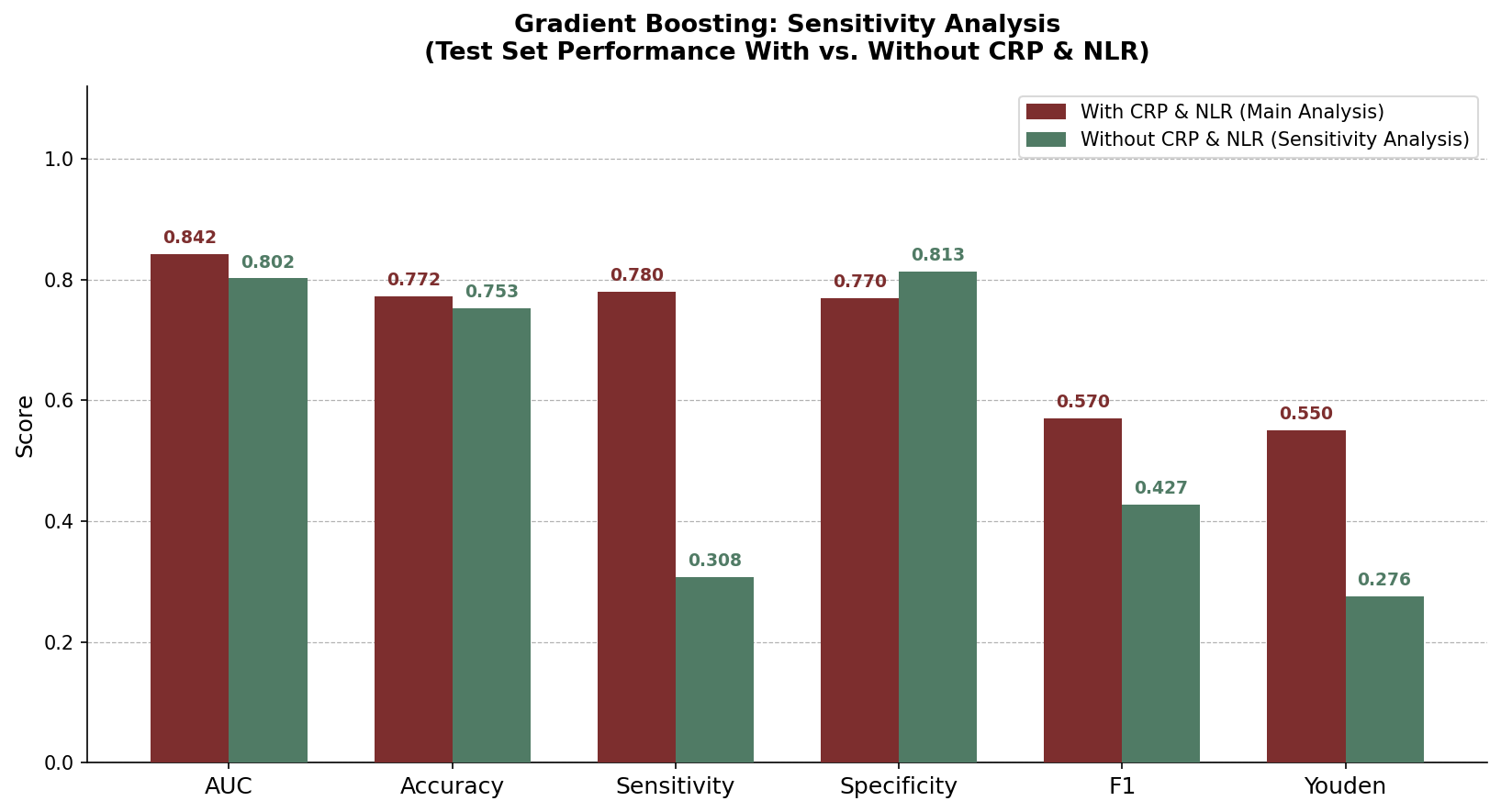


1.6 Methodological Explanation for Handling Class Imbalance


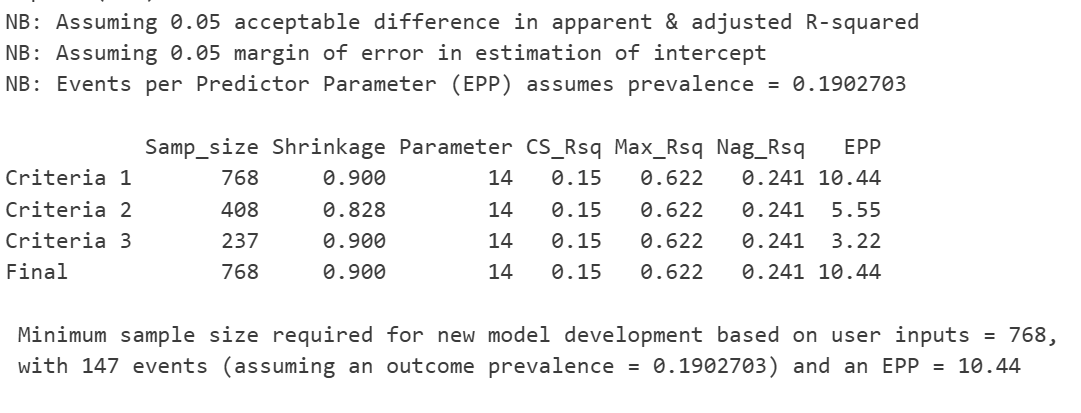


Sample size estimation for prediction model development was performed using the method proposed by Riley et al., implemented via the **pmsampsize** package in R (version 4.2.2). Based on the observed outcome prevalence of 19.0% (176/925), 14 candidate predictor parameters, a conservative anticipated Cox–Snell R² of 0.15, and a shrinkage factor threshold of 0.9, the minimum required sample size was estimated to be 768 participants with at least 147 outcome events (EPP = 10.44). This requirement was derived primarily from the criterion of minimising overfitting (shrinkage ≥ 0.9). The final study sample of 925 participants (176 outcome events, EPP = 12.57) exceeded the minimum requirement, indicating adequate sample size for model development[1].

[1]Reference: Riley RD, et al. Minimum sample size for developing a multivariable prediction model: Part I – Continuous outcomes. Statistics in Medicine. 2019;38(7):1262–1275.
